# Modulations of static and dynamic functional connectivity among brain networks by electroacupuncture in post-stroke aphasia

**DOI:** 10.3389/fneur.2022.956931

**Published:** 2022-12-01

**Authors:** Minjie Xu, Ying Gao, Hua Zhang, Binlong Zhang, Tianli Lyu, Zhongjian Tan, Changming Li, Xiaolin Li, Xing Huang, Qiao Kong, Juan Xiao, Georg S. Kranz, Shuren Li, Jingling Chang

**Affiliations:** ^1^Department of Neurology, Dongzhimen Hospital, Beijing University of Chinese Medicine, Beijing, China; ^2^Key Laboratory of Chinese Internal Medicine Ministry of Education, Beijing University of Chinese Medicine, Beijing, China; ^3^Institute for Brain Disorders, Beijing University of Chinese Medicine, Beijing, China; ^4^Department of Rehabilitation Sciences, The Hong Kong Polytechnic University, Hong Kong, Hong Kong SAR, China; ^5^The State Key Laboratory of Brain and Cognitive Sciences, The University of Hong Kong, Hong Kong, Hong Kong SAR, China; ^6^Department of Psychiatry and Psychotherapy, Comprehensive Center for Clinical Neurosciences and Mental Health, Medical University of Vienna, Vienna, Austria; ^7^Division of Nuclear Medicine, Department of Biomedical Imaging and Image-Guided Therapy, Medical University of Vienna, Vienna, Austria

**Keywords:** electroacupuncture, brain networks, post stroke aphasia, functional connectivity, psychophysiological interaction analysis, independent component analysis

## Abstract

**Introduction:**

Post-stroke aphasia (PSA) is a language disorder caused by left hemisphere stroke. Electroacupuncture (EA) is a minimally invasive therapeutic option for PSA treatment. Tongli (HT5) and Xuanzhong (GB39), two important language-associated acupoints, are frequently used in the rehabilitation of patients with PSA. Preliminary evidence indicated functional activation in distributed cortical areas upon HT5 and GB39 stimulation. However, research on the modulation of dynamic and static functional connectivity (FC) in the brain by EA in PSA is lacking.

**Method:**

This study aimed to investigate the PSA-related effects of EA stimulation at HT5 and GB39 on neural processing. Thirty-five participants were recruited, including 19 patients with PSA and 16 healthy controls (HCs). The BOLD signal was analyzed by static independent component analysis, generalized psychophysiological interactions, and dynamic independent component analysis, considering variables such as age, sex, and years of education.

**Results:**

The results revealed that PSA showed activated clusters in the left putamen, left postcentral gyrus (PostCG), and left angular gyrus in the salience network (SN) compared to the HC group. The interaction effect on temporal properties of networks showed higher variability of SN (*F* = 2.23, positive false discovery rate [pFDR] = 0.017). The interaction effect on static FC showed increased functional coupling between the right calcarine and right lingual gyrus (*F* = 3.16, pFDR = 0.043). For the dynamic FC, at the region level, the interaction effect showed lower variability and higher frequencies of circuit 3, with the strongest connections between the supramarginal gyrus and posterior cingulum (*F* = 5.42, pFDR = 0.03), middle cingulum and PostCG (*F* = 5.27, pFDR = 0.036), and triangle inferior frontal and lingual gyrus (*F* = 5.57, pFDR = 0.026). At the network level, the interaction effect showed higher variability in occipital network–language network (LN) and cerebellar network (CN) coupling, with stronger connections between the LN and CN (*F* = 4.29, pFDR = 0.042). Dynamic FC values between the triangle inferior frontal and lingual gyri were anticorrelated with transcribing, describing, and dictating scores in the Chinese Rehabilitation Research Center for Chinese Standard Aphasia Examination.

**Discussion:**

These findings suggest that EA stimulation may improve language function, as it significantly modulated the nodes of regions/networks involved in the LN, SN, CN, occipital cortex, somatosensory regions, and cerebral limbic system.

## Introduction

Post-stroke aphasia (PSA) is a clinical syndrome caused by a left hemisphere stroke that results in the loss of language skills and consequently has an impact on daily life (1). Even mild aphasia can have a negative effect on functional outcomes, such as mood, quality of life, and ability to return to work (2). Language is a critical cognitive skill supported by large-scale brain networks (3). In addition to the direct effect of focal lesions on important cortical regions, damage to other remote areas within language networks and non-language-specific networks also leads to the occurrence and development of aphasia (4, 5). It has been suggested that focal stroke lesions can affect language comprehension by altering the functional connectivity (FC) of multiple networks and subnetworks in PSA (6–8).

Acupuncture is an ancient Chinese treatment that has been systematically used for 2000 years (9). It is rapidly gaining recognition for its therapeutic properties in the treatment of PSA and several other neurological conditions (10–13). Electroacupuncture (EA) is a modern form of acupuncture that features a small current passing between pairs of acupuncture needles. To observe the immediate effect of acupuncture on brain activity, several recent studies have applied EA during functional magnetic resonance imaging (fMRI) (14–16). Block design is one of the most commonly used scanning methods for exploring the potential mechanisms of EA in fMRI studies (17–20). Tongli (HT5) and Xuanzhong (GB39) are important language-implicated acupoints used in Chinese medicine. They are commonly used in PSA rehabilitation therapy because they are tailored to language-processing systems. Previous fMRI studies of EA treatment demonstrated that stimulation at HT5 and GB39 resulted in activation of language regions and the somatosensory cortex in both cerebral hemispheres and that it might modulate speech function through effects on brain networks in healthy individuals (21–23).

It is worth noting that the emergence of acupuncture as a treatment stem from its function in patients rather than in healthy people (24). Modulation of information processing in the brain through acupuncture may differ between patients with PSA and healthy individuals and occurs mainly in disorder-related areas (24). A direct comparison of the effect on neural networks between patients with PSA and healthy individuals involved in the processing of EA stimulation at HT5 and GB39 is virtually absent from the present research. Moreover, although the aforementioned studies have focused on localizing a series of brain regions showing activation patterns during the processing of EA stimulation in healthy individuals, a rapidly growing body of neuroimaging literature suggests that the effect of acupuncture in patients with stroke may be attributed to modulation of disrupted patterns of the whole-brain network rather than in one or two confined brain regions (25, 26).

Therefore, this study aimed to explore and compare how EA stimulation modulates FC and temporal properties in patients with PSA and healthy individuals. To address this question, independent component analysis (ICA) and general psychophysiological interaction (gPPI) were used to reveal static and dynamic FC changes at the levels of the region of interest (ROI) and large-scale network.

## Materials and methods

### Participants and ethics statements

Based on previous study results, 35 right-handed participants were recruited between August 2013 and February 2021, including 19 patients with PSA (33–78 years, 13 men) and 16 demographically matched healthy volunteers (24–63 years, right-handed, eight men). Patients with PSA were recruited from the Department of Neurology at Dongzhimen Hospital with complaints of language disorders. Healthy controls (HCs) were recruited from the local community.

**Figure 1:**
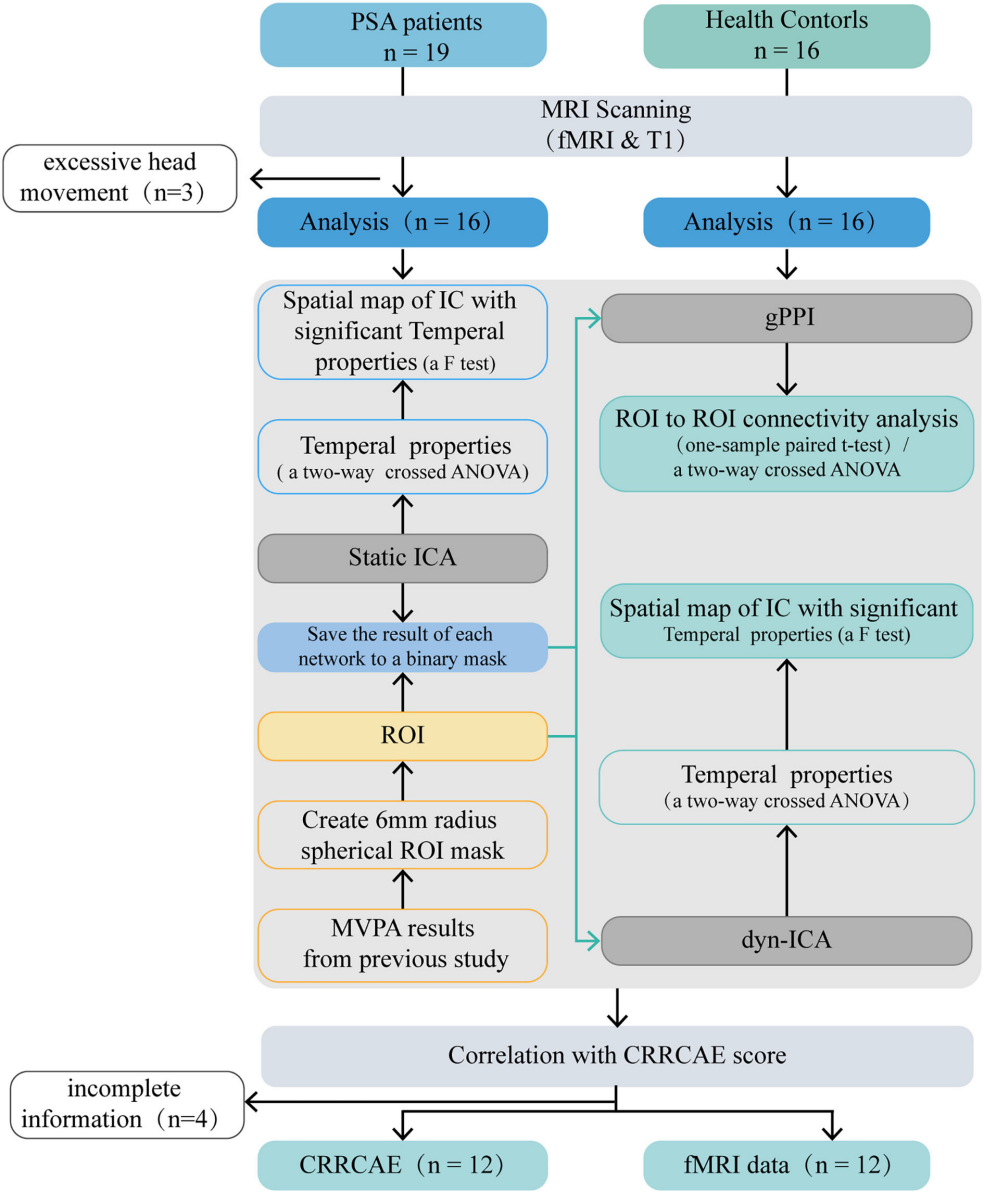
Flow diagram. PSA, post stroke aphasia; gPPI, general psychophysiological interaction; dyn-ICA, dynamic independent component analysis; static ICA, static independent component analysis; CRRCAE, Chinese Rehabilitation Research Center for Chinese Standard Aphasia Examination; ANOVA, analysis of variance; ROI, region of interest; MVPA, multi-voxel pattern analysis.

The Medical Research Ethics Committee of Dongzhimen Hospital (reference number: ECPJ-BDY-2015-04) approved this study, and all participants provided written consent to participate. An overview of the study design is shown in Figure 1.

### Clinical evaluation and speech and language testing

Demographic factors, sex, age, handedness, years of education (Edu), and scores on the Boston Diagnostic Aphasia Examination were reviewed for each patient. Twelve patients with motor aphasia were diagnosed by the Chinese Rehabilitation Research Center for Chinese Standard Aphasia Examination (CRRCAE), a battery of language tests designed according to the Mandarin rules. As a result of testing the reliability and validity of the CRRCAE in a prior study, it was found to have good sensitivity and reliability and may be used as a quantitative table for the diagnosis of aphasia in Mandarin speakers (27, 28). Thirty subscales including comprehension, speaking, writing, calculation, copy, and repeating comprise the scale (28).

### Stimuli and scanning procedure

#### Acupuncture procedures and needling sensation recording

**Figure 2:**
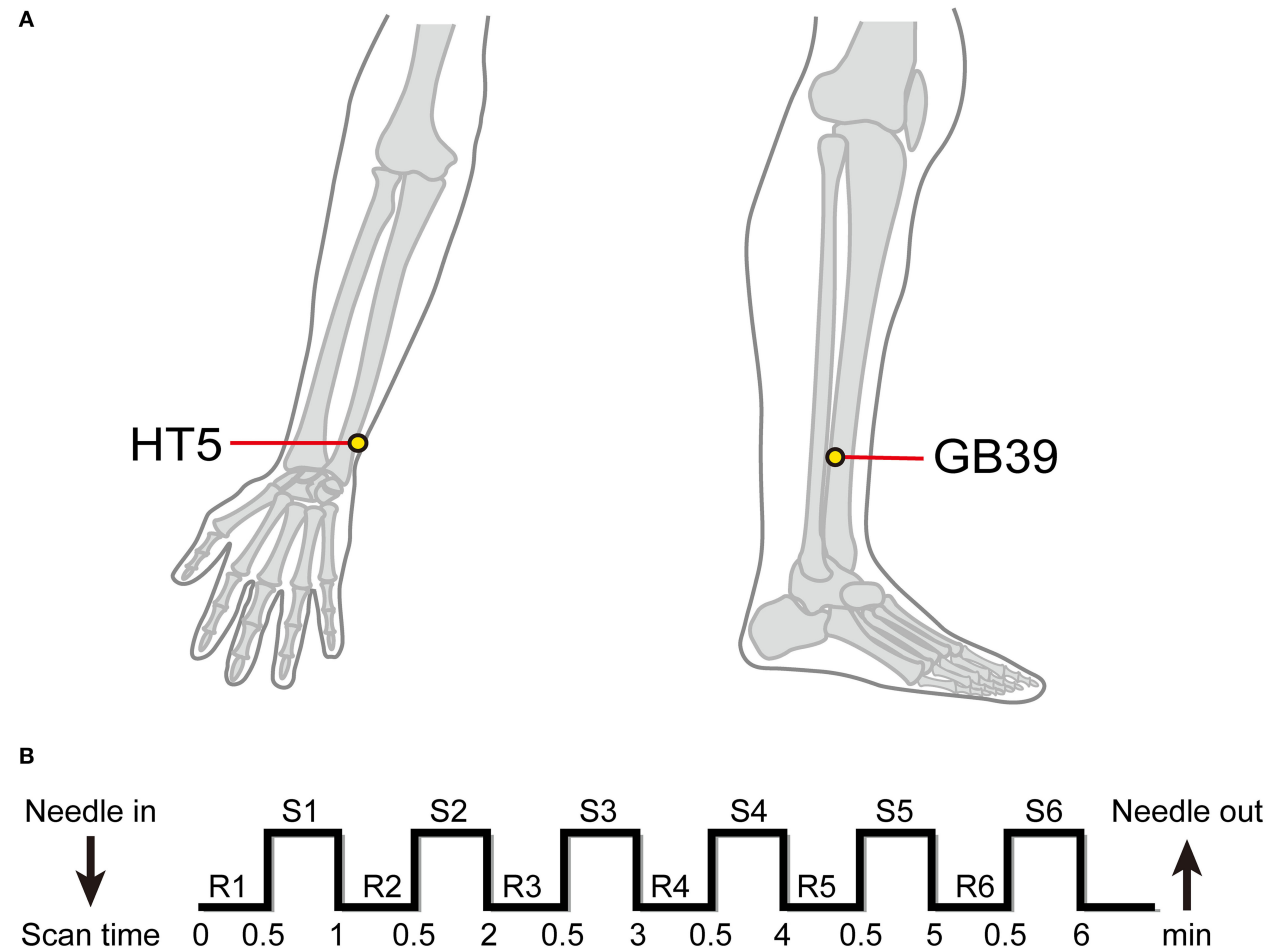
Location of acupuncture points and electroacupuncture stimulation procedure. **(A)** Location of HT5 and GB39. **(B)** n-Block design of task-based fMRI. R, rest; S, stimulation.

Both the PSA and HC groups received EA stimulation at the HT5 and GB39 acupoints during fMRI acquisition. Before the start of the scan, the participants positioned themselves on the fMRI scanner bed on their backs, and needles were placed at the GB39 and HT5 acupoints. According to the “Name and Location of Acupoints” (GB/T 12346-2006), two acupoints were located on both sides. HT5 was situated radially to the flexor carpi ulnaris tendon on the anteromedial side of the forearm, 33 mm proximal to the palmar wrist crease, with insertion depths ranging from 10 to 30 mm. GB39 was needled at an insertion depth of 33 mm, 100 mm above the external malleolus tip, on the anterior fibula border (Figure 2A). A professional acupuncturist performed acupuncture. All participants reported their experience (“Deqi”) with acupuncture stimulation. Deqi featured aching, pressure, heaviness, fullness, and numbness among other feelings (23–29). The acupuncturist used 0.40 × 40-mm sterile silver acupuncture needles (Guizhou, China) with the EA technique. Han's acupoint nerve stimulator (model LH-202H) was situated outside the fMRI room, with one end of the acupoint wire linked to the acupuncture needle handle of the HT5 acupoint, and the other end connected to the acupuncture needle handle of the GB39 acupoint. The EA frequency was 2 Hz, and the electric current was 2 mA. As previously mentioned, the stimulation waveform is the dilatational wave (23).

#### fMRI scanning

All fMRI data were acquired using the 3 Tesla Siemens MRI scanner (Erlangen, Germany) at Dongzhimen Hospital. A high-resolution T1-weighted structural image was obtained using an isotropic multi-echo magnetization-prepared rapid acquisition sequence (repetition time: 1900 ms, echo time: 2.13 ms, field of view: 250 mm, flip angle: 90°, voxel size: 1.0 × 1.0 × 1.0 mm, slice thickness: 1.0 mm, matrix: 256 × 256, number of slices: 176). Functional images were acquired using a single shot gradient-recalled echo planar imaging sequence (31 interleaved axial slices, repetition time: 2,000 ms, echo time: 30 ms, flip angle: 90°, field of view: 225 × 225 mm^2^, interslice gap: 0.7 mm, matrix: 64 × 64 mm^2^, slice thickness: 3.5 mm, voxel size: 3.5 × 3.5 × 3.5 mm^3^). The fMRI experimental design was a classic resting/stimulation block design divided between a 30-s resting interval and a 30-s stimulation period; such blocks were repeated for six cycles, six acupuncture blocks, and six rest blocks out of a total of 12 blocks that were completed in one session (30) (Figure 2B).

### Data processing

The Resting-State fMRI Data Analysis Toolkit (REST plus, version 1.24; http://www.restfmri.net/forum/RESTplus) (31) and task-fMRI images were preprocessed using the Statistical Parametric Mapping 12 software suite (SPM12, http://www.fil.ion.ucl.ac.uk/spm). First, we discarded the first 10 volumes for each participant to avoid the negative impact of magnetic disequilibrium on data quality. Subsequently, all functional images were preprocessed [slice acquisition time, head motion, and a 6-mm Gaussian kernel with full width at half maximum (FWHM) smoothing]. All data had head motions < 3 mm or 3°. Next, using Advanced Normalization Tools (ANTS1.9, https://www.nitrc.org/projects/ants), the functional images were spatially normalized to the Montreal Neurological Institute space in the Linux platform. Temporal filtering was also applied, which allowed a frequency band of 0.01–0.1 Hz to pass. Subsequently, each participant was subjected to linear detrending to remove any residual effect of low-frequency drift or high-frequency physiological noise.

### Statistical analysis

All statistical analyses were performed using the CONN toolbox (32). We examined the effects of EA on FC using a condition main effect (group: any effect, condition: EA stimulation > rest at baseline), the PSA-related effect using a group main effect (group: PSA-HC, condition: any effect), and the interaction effect using a two-way crossed analysis of variance (ANOVA), with years of Edu, age, and sex as covariates, with a within-subjects factor (condition: EA stimulation vs. rest at baseline) and a between-subjects factor (group: patients with PSA vs. HCs). At a threshold of *p* < 0.05, the false discovery rate-corrected results were considered statistically significant.

#### Static-independent component analysis

Implemented in CONN 18b, static-ICA employs the Fast-ICA method and GICA3 back projection to estimate independent components (ICs) and subject-level spatial map estimation (33). Twenty estimated components were chosen (34). The spatial maps and time courses were normalized to *z*-scores for future analyses.

##### Spatial components

**Figure 3:**
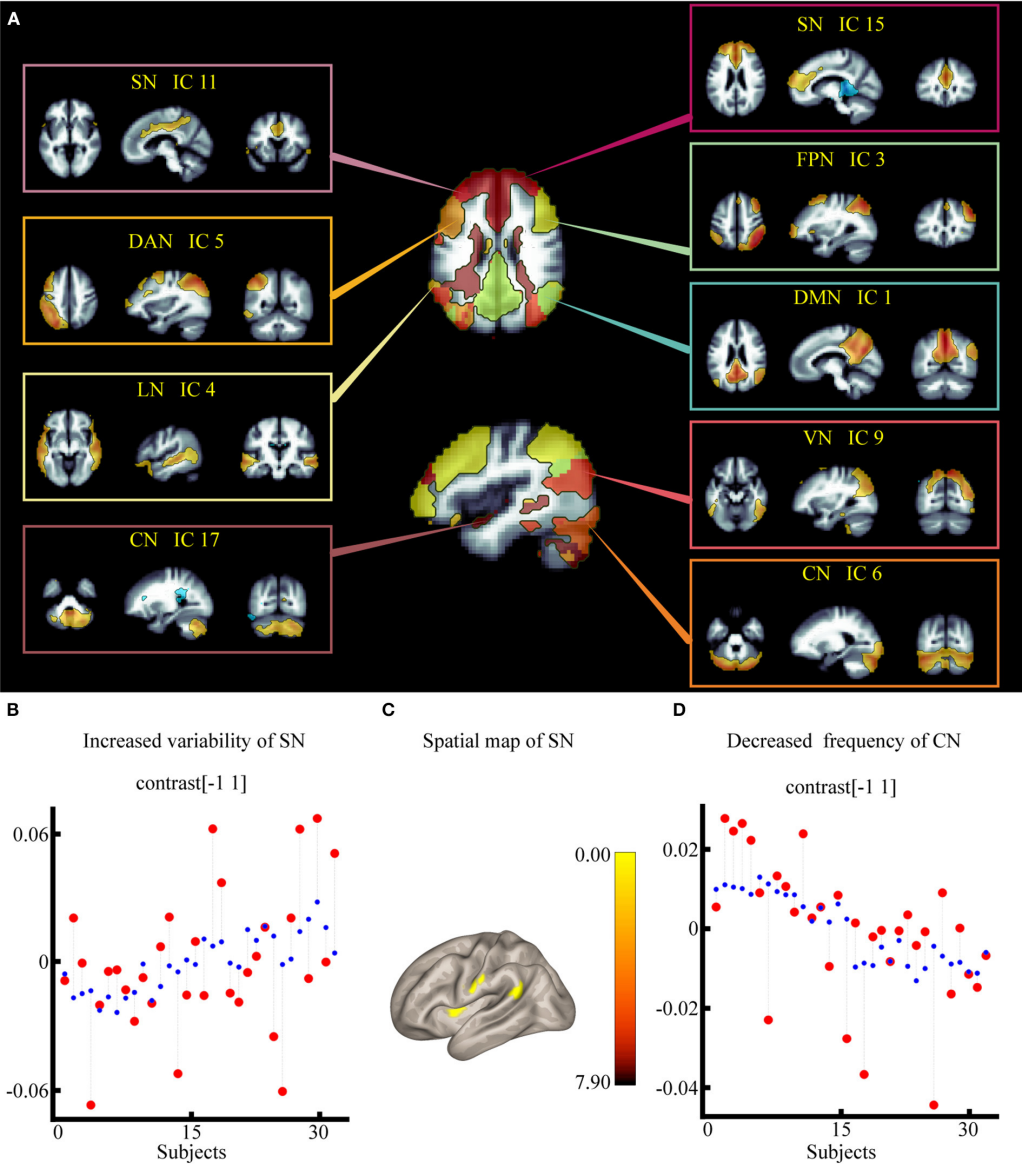
Effect of electroacupuncture stimulation on static independent component analysis. **(A)** Nine spatial maps of intrinsic resting-state networks in 32 subjects. **(B)** Changes in temporal properties varies of SN within each group and condition. **(C)** Spatial map of SN for the contrast of post stroke aphasia group vs. healthy control group. **(D)** Changes in temporal properties varies of VN within each group and condition. DMN, default mode network; SMN, sensorimotor network; VN, visual network; SN, salience network; DAN, dorsal attention network; FPN, frontoparietal network; LN, language network; CN, cerebellar network.

The dorsal attention network (DAN), default mode network (DMN), sensorimotor network (SMN), salience network (SN), visual network (VN), frontoparietal network, language network (LN), and cerebellar network (CN) were identified within each component using spatial sorting and a correlational spatial match-to-template approach (spatial correlation and spatial overlap of suprathreshold areas/dice coefficient) (35). Nine components were chosen from a total of 20 ICs to represent the brain networks (Figure 3A). Among them, instead of calling network 9 the VN, we chose to call it the occipital network, as there was no visual stimulus in this study. From now on, we shall refer to components as “networks” and we will identify components by referring to their label (e.g., DMN); component descriptions are listed in Supplementary Table 1. For the spatial map, we examined how the PSA-related network structure differed between the two groups using a voxel-wise F-test (group main effect), as in previous studies (36).

##### Temporal components

First, for each individual, a set of nine beta coefficients for each of the two condition regressors was derived, indicating the degree to which the task regressor relates to a specific network. Then, to verify the interaction effect (group × condition) on BOLD signal fluctuations in each brain network, we performed a two-way crossed ANOVA test for the nine remaining networks using the temporal properties implemented in the CONN toolbox.

#### GPPI-based FC analyses

Unsmoothed (37) but preprocessed (refer to data processing) fMRI data were inputted into the CONN toolbox for additional preprocessing and gPPI analysis (38). Before conducting the gPPI analysis, we used a component-based noise correction method to remove artifacts from the fMRI data. To investigate how EA stimuli alter brain functional architecture at the ROI (Supplementary Figure 1) and large-scale network levels (static ICA results), we used gPPI analysis according to Lee Masson et al. (23–39). The regression model used in the gPPI analysis was as follows: *Y* = *X*_1_ × *β* + *X*_2_ × *βG* + *error*, where *Y* = time-series BOLD response of the seed, *X*_1_ = the hemodynamic response function (HRF) convolved gPPI terms (i.e., time-series BOLD response of the seed × the psychological regressor as dictated by onset and duration of EA stimulus), *β* = the strength of FC (i.e., the beta estimates of gPPI terms), *X*_2_ = *X*_1_ × HRF convolved psychological regressor × covariates of no interest (age, Edu, and sex regressors), and *βG* = the beta estimates of the HRF-convolved time-series BOLD response of the seeds. Individual results were transformed into *z*-scores using the Fisher *z*-transformation for the group-level analysis. First, we evaluated how FC was modulated by the stimuli in each group using a one-sample paired *t*-test. Second, we used a two-way crossed ANOVA to assess the interaction effect on FC strength (40).

#### Dynamic independent component analysis

Dynamic connectivity measures were used to examine and characterize the sources of FC variability. Dyn-ICA runs an ICA on the connection time series and returns ICs that most accurately represent FC modulation over time (41, 42). For first-level signal processing, Dyn-ICA matrices (circuits) are created using dynamic connectivity measures. Seeds used to build the matrices were obtained from our previous multi-voxel pattern analysis results (23) (Supplementary Figure 1) and static ICA results in this study (Figure 3A). Then, the aggregated data were divided into 12 circuits using Dyn-ICA with 30 smoothing kernels, in line with a previous study's method (43). This processing produces multiple outputs, including the individual subject-level matrices gamma and the variability and frequency of the dynamic circuit time series. For the second level, we statistically evaluated (1) the frequency of each component (temporal component time-series frequency averaged across all participants) and (2) the variability of each component (temporal component time-series standard deviation averaged across all participants), and the spatial properties (defined as gPPI interaction terms). A two-way crossover ANOVA test was used to assess the interaction effect of temporal data on the frequency and variability of dynamic circuits with Edu, age, and sex as covariates of no interest. Finally, a main effect of condition was performed to assess the EA stimulus-dependent spatial characteristics of circuits with significant temporal properties.

### Correlation analysis between behavioral data and fMRI data during EA stimulation

To evaluate the correlation between brain network modulation during EA and behavioral data (CRRCAE scores) in patients with PSA, we conducted a rank correlational analysis. Please note that the correlation analyses were exploratory; thus, a *p*-value < 0.05 with no corrections for multiple comparisons was implemented, in line with a previous study's methods (44). Correlation analysis was performed using the R software package (The R Project for Statistical Computing, Vienna, Austria).

## Results

### Clinical characteristics and indices

Detailed clinical characteristics and indices are summarized in Table 1. Patients with PSA and the HCs exhibited no significant differences in age or sex, but they showed significant differences in Edu (*p* ≤ 0.001).

**Table 1:** Demographic data and clinical characteristics of the PSA and HCs groups.

**Characteristics**	**PSA patients**	**HCs**	***P*** **-values**
Gender, female/male, *n*	5:11	3:13	0.41
Handedness, right/left, *n*	16:0	16:0	1.00
Age, years, mean ± SD	58.56 ± 12.14	42.81 ± 13.38	0.16
Edu, years mean ± SD	12 ± 2.52	15.25 ± 4.22	0.00
Listening mean ± SD	25 ± 9		
Repeating mean ± SD	20 ± 7		
Speaking mean ± SD	11 ± 10		
Reading aloud mean ± SD	17 ± 7		
Reading comprehension mean ± SD	21 ± 10		
Transcribing mean ± SD	3 ± 4		
Describing mean ± SD	2 ± 4		
Dictating mean ± SD	1 ± 2		
Calculating mean ± SD	3 ± 4		
BDAE, mean	2		

Edu, years of education; EA, electroacupuncture; LN, language network; CN, cerebellar network; SN, salience network; ROI, region of interest; SMG, supramarginal gyri; PostCG, postcentral gyri; PSA, post stroke aphasia; HC, healthy control; DMN, default mode network; positive false discovery rate, p-FDR; MNI, montreal neurological institute; R, right; L, left.

### Spatial map and temporal properties of each IC

#### Varied interaction effect of temporal properties

According to the results of the two-way crossed ANOVA test, there was a significant effect on the degree of synchronization, including increased variability of SN [*F* = 2.23, positive false discovery rate (pFDR) = 0.017] (Figure 3B) and lower frequency of CN [*F* = −2.93, pFDR = 0.006] (Figure 3D; Supplementary Table 1).

#### Spatial map for the comparison of the PSA and HC groups

According to the results of the group main effect, different contributions from certain brain regions in the nine networks were detected. We found that the PSA group showed activated clusters in the left putamen, left postcentral gyrus (PostCG), and left angular gyrus (AG) in the SN compared to the HC group. No significant differences were observed in CN. Results are summarized in Figure 3C and Table 2.

**Table 2:** Spatial map of SN for the contrast of PSA group vs. HC group.

**Regions**	**Clusters**			**Size**	**Side**	***F***	***p*** **-FDR**
	***x***	***y***	***z***				
Putamen	−30	−06	06	54	left	−3.92	0.003
AG	−57	−51	15	53	left	−4.45	0.003
PostCG	−60	−15	30	39	left	−4.00	0.010

Edu, years of education; EA, electroacupuncture; LN, language network; CN, cerebellar network; SN, salience network; ROI, region of interest; SMG, supramarginal gyri; PostCG, postcentral gyri; PSA, post stroke aphasia; HC, healthy control; DMN, default mode network; positive false discovery rate, p-FDR; MNI, montreal neurological institute; R, right; L, left.

### Static FC during EA stimulation

#### Interaction effect on static FC

**Figure 4:**
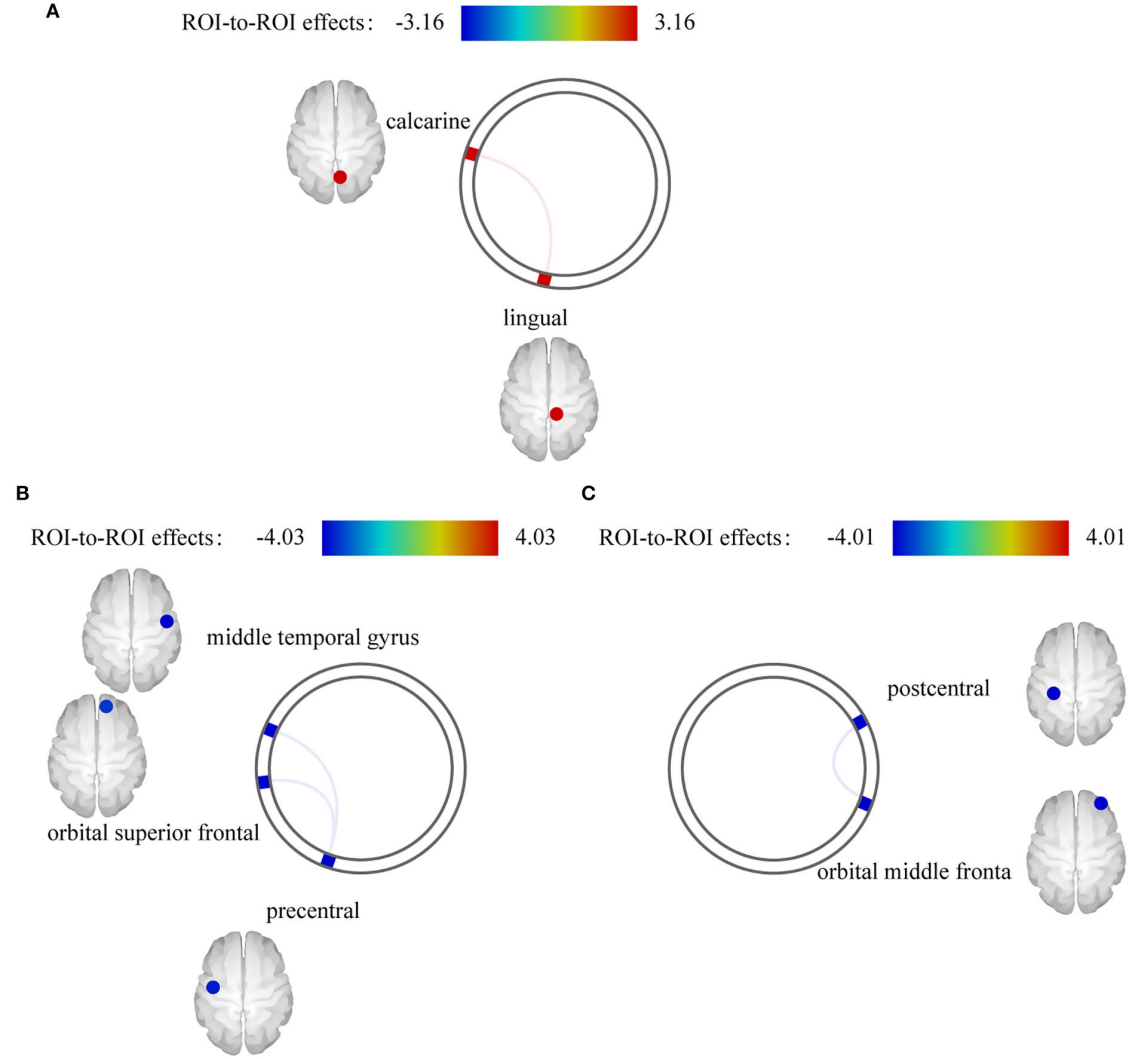
Effect of electroacupuncture (EA) stimulation on static functional connectivity (FC). **(A)** Interaction effect on static FC. **(B)** EA stimulus-dependent changes in FC for the contrast of EA stimulation vs. rest at baseline in post stroke aphasia group. **(C)** EA stimulus-dependent changes in FC for the contrast of EA stimulation vs. rest at baseline in healthy control group.

We found an interaction effect on functional coupling between the right calcarine and right lingual gyrus [*F*_(14, 27)_ = 3.16, pFDR = 0.043] (Figure 4A; Table 3). However, no significant differences were observed at the network level.

**Table 3:** Significant static FC values during EA stimulation.

**Regions**	**Side**	**MNI coordinates**			**Regions**	**Side**	**MNI coordinates**			***F***	***p*** **-FDR**
		***x***	***y***	***z***			***x***	***y***	***z***		
**EA stimulation** **>** **rest at baseline in PSA**
Precentral	L	−44	−9	41	Middle temporal gyrus	R	52	−4	−20	−4.03	0.024
					Orbital superior frontal	R	14	55	−17	−3.35	0.048
**EA stimulation** **>** **rest at baseline in HC**
Postcentral	L	−32	−31	67	Orbital middle frontal	R	37	54	−5	−4.01	0.025
**Group** **×condition interaction effect**
Calcarine	L	−3	−93	11	Lingual	R	12	−39	0	3.16	0.043

Edu, years of education; EA, electroacupuncture; LN, language network; CN, cerebellar network; SN, salience network; ROI, region of interest; SMG, supramarginal gyri; PostCG, postcentral gyri; PSA, post stroke aphasia; HC, healthy control; DMN, default mode network; positive false discovery rate, p-FDR; MNI, montreal neurological institute; R, right; L, left.

#### Changes in FC between EA stimulation and rest in each group

We also separately identified the FCs between ROIs during EA stimulation in each group. EA stimulation reduced FC between the left precentral and right middle temporal gyrus [*F*_(4,13)_ = −4.03, pFDR = 0.024] and right orbital superior frontal gyrus [*F*_(4,11)_ = −3.35, pFDR = 0.048] in PSA (Figure 4B). Additionally, ROI-based gPPI analysis of the HC group revealed that EA stimulation reduced FC between the left postcentral and right orbital middle frontal regions [*F*_(3,5)_ = −4.01, pFDR = 0.025], compared with the rest condition (Figure 4C). The results are presented in Table 3. Network-based gPPI analysis revealed no significant differences in either HC groups or PSA groups.

### Dynamic FC during EA stimulation

#### Results of the interaction effects at the network level

**Figure 5:**
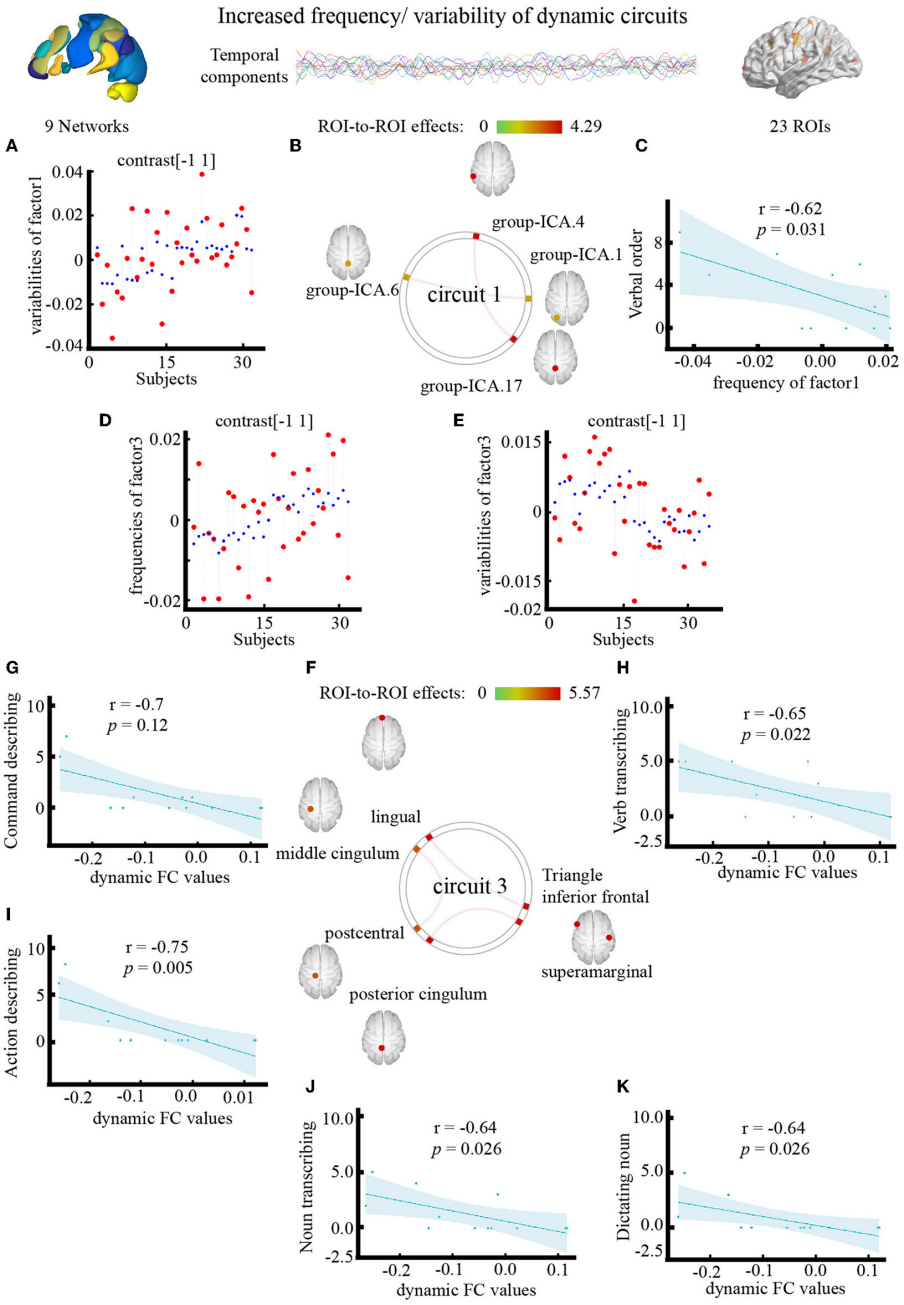
Effect of electroacupuncture (EA) stimulation on dynamic independent component analysis. **(A)** Changes in temporal properties varies of circuit1. **(B)** Spatial map of circuit1 for the contrast of condition main effect. **(C)** Correlation between frequency of circuit1 and clinical parameter (CRRCAE score). **(D,E)** Changes in temporal properties varies of circuit3. **(F)** Spatial map of circuit3 for the contrast of condition main effect. **(G–K)** Correlation between clinical parameter (CRRCAE score) and dynamic FC values of triangle inferior frontal and lingual. CRRCAE, Chinese Rehabilitation Research Center for Chinese Standard Aphasia Examination.

The outlook of the 12 components is shown in Supplementary Figure S2. Patients with PSA had higher variability of occipital network LN and CN coupling (Figure 5A), with stronger connections between the LN and CN (*F* = 4.29, pFDR = 0.042, uncorrected *p* = 0.0053), DMN and CN (*F* = 4.28, pFDR = 0.043, uncorrected *p* = 0.005) (Figure 5B; Table 4).

**Table 4:** PSA-related differences in dynamic functional connectivity during EA stimulation.

**Components**	**Temporal properties**	***F***	***p*** **-FDR**	**Spatial properties**				***F***	***p*** **-FDR**
				**Regions**	**Side**	**Regions**	**Side**		
**ROI level**
Circuit3	Frequency	2.28	0.030	Lingual	R	Triangle inferior frontal gyri	L	5.57	0.026
				SMG	R	Posterior cingulum gyri	L	5.42	0.031
Circuit3	Variability	−2.23	0.034	PostCG	L	Middle cingulum gyri	L	5.27	0.036
**Network level**
Circuit1	Variability	2.34	0.026	DMN		CN		4.28	0.043
				LN		CN		4.29	0.042

Edu, years of education; EA, electroacupuncture; LN, language network; CN, cerebellar network; SN, salience network; ROI, region of interest; SMG, supramarginal gyri; PostCG, postcentral gyri; PSA, post stroke aphasia; HC, healthy control; DMN, default mode network; positive false discovery rate, p-FDR; MNI, montreal neurological institute; R, right; L, left.

#### Results of the interaction effects at the ROI level

The outlook of the 12 components is shown in Supplementary Figure S3. Patients with PSA had higher frequencies (Figure 5D) and lower variability (Figure 5E) of circuit 3, which connects the occipital, limbic, and somatosensory systems, with the strongest connections between the triangle inferior frontal and lingual gyri (*F* = 5.57, pFDR = 0.026, uncorrected *p* = 0.001), supramarginal and posterior cingulum gyri (*F* = 5.42, pFDR = 0.03, uncorrected *p* = 0.001), and middle cingulum and postcentral gyri (*F* = 5.27, pFDR = 0.036, uncorrected *p* = 0.001) (Figure 5F; Table 4). The frequency of factor 3 was directly anticorrelated with the verbal order score (uncorrected *p* = 0.031) (Figure 5C), and the FC values between the triangle inferior frontal and lingual gyri were directly anticorrelated with the noun transcribing score (uncorrected *p* = 0.026), verb transcribing score (uncorrected *p* = 0.022), command describing score (uncorrected *p* = 0.011), action description score (uncorrected *p* = 0.005), and dictating noun score (uncorrected *p* = 0.026) (Figures 5G–K).

## Discussion

This study aimed to calculate the differences in EA stimulus-dependent changes in brain connectivity patterns between patients with PSA and healthy individuals. The interaction between EA stimulation and disease was examined using static ICA, gPPI and Dyn-ICA, with age, sex, and Edu as covariates, similar to a previous study's methods (45). By using static ICA and dynamic ICA approaches, we discovered commonalities with previous studies of acupuncture neuroimaging mechanisms for PSA, as well as some prominent points/networks generated from newer methodologies. The present study is the first to identify a PSA-related neuroimaging mechanism of EA stimulation at HT5 and GB39 both at the ROI and the network level. Our research showed that EA stimulation dramatically changed the nodes of the regions/networks involved in the LN, SN, CN, occipital cortex, somatosensory regions, and cerebral limbic system. Our previous research on the effects of EA on PSA was expanded by these results.

### Static ICA

Recently, it has been acknowledged that stroke pathology often affects large-scale functional network structures rather than lesions (46). Based on previous studies, the therapeutic effects of acupuncture extend to the entire brain area and even to the brain network (23). Such influences cannot be calculated using a univariate approach (47) but can be solved using an ICA approach. First, ICA can find functionally independent, potentially spatially overlapping functional networks without the need for prior information of the task paradigm (48). Second, it enables researchers to assess the effects of EA on wider functional networks in patients with PSA. Third, mean activity values within networks as well as frequency and variability distributions could be computed, and the impacts of EA on different portions of a particular network in individual patients contributed to an overall change in score, making this technique more resilient to the impact of varying structural lesions. In the present study, the mean activity levels of left AG and left PostCG in the SN were higher in patients with PSA than in HCs, and the temporal distributions showed significantly higher SN variability, same as the previous study (7). There is abundant evidence in the literature that self-generated speech activates the SN, and the SN had been provided to have a directly correlated with residual language performance in patients with PSA (49). The AG is a region associated with phonological deficits (50). The fact that only individuals with motor aphasia were included may help to explain this. Thus, our findings suggest that EA causes the SN network to transmit more information in motor aphasia.

### Static FC analysis

Studies on resting-state connections in healthy persons assume that positive connectivity represents integration and coordination between different brain regions, whereas negative connectivity represents separated or conflicting systems (51). First, we computed the effect of the stimulation condition against the rest of the baseline conditions in each group. Second, we calculated the interactions between the groups and conditions. Our sample's modifications were primarily connected to the somatosensory, language, and occipital cortices. These regions were previously shown to be associated with verb learning and visual word processing (52), and the integration of the visual and sensorimotor systems sustains action naming (53). Our results showed that a crucial neuronal signature underlying EA stimulation processing in PSA may be a change in communication across key regions involved in vision and naming.

### Dyn-ICA analysis

These theories assume that FC is “static,” while mounting data support the idea that FC is dynamic rather than static (54). Dyn-ICA can examine time-varying and dynamic extensions of component analysis, which can be accomplished by using a classic component analysis within a sliding window setting (55), and isolate the signal from the noise and boost the sensitivity to identify individual differences (56). Previous studies have used Dyn-ICA to observe the pathological characteristics of diseases (43), and we are the first to use Dyn-ICA to study the mechanism of the effect of acupuncture. Similarly, our studies have clearly shown that Dyn-ICA increases sensitivity to variations between individuals or conditions (56). We used Dyn-ICA to incorporate temporal information, such as frequency and variability, while maintaining the spatial identification of traditional FC at the ROI and network level and arrived at two primary conclusions. First, at the ROI level, PSA-related dynamic connection showed a higher frequency of occipital cortex-limbic system-sensorimotor system connections, and their variability decreased across different dynamic factors. The structure of the FC map showed enhanced connectivity among the occipital, limbic, and sensorimotor system cortices. The higher FC values between the triangle inferior frontal and lingual gyri in the dynamic condition main effect correlated with worse transcribing, describing, and dictating scores. The same results can be found in a previous static analysis of the effect of acupuncture with sham acupuncture as a control (26), but not in a dynamic framework, to the best of our knowledge. Second, perhaps more importantly, at the network level, our results showed that PSA-related dynamic connections between the CN, DMN, and LN are more frequent during EA stimulation than rest at baseline. We found that EA at HT5 and GB39 changes the synchronization between the cerebellum and cerebrum, which is consistent with findings of a previous study (57). The DMN is a collection of areas that are more active at rest than when performing a task (58). These regions are believed to play a role in internal or low-level attentional mechanisms (59). Considerable evidence has demonstrated the essential involvement of the CN in a variety of language functions (60), and CN maps to cerebral association networks have already been reported to correlate with cognitive function (61). Our findings suggest that an aspect of the PSA treatment effect of EA stimulation may be a time-dependent connection between these networks of language function correlations.

### Limitations of the present study

This study has some limitations. First, the lesioned voxels can affect fMRI preprocessing and results. However, to the best of our knowledge, there is no accepted standard procedure for the treatment of stroke lesions. We did not mask the lesion, considering that manual mask procuring is time-consuming and subjective (62). The quality of the segmentation and normalization does not seem to be affected in this study, and by adopting the ICA method, some effects of stroke lesions may be offset (56). However, we must admit that there is still more to be done in the future to address this problem. Second, pain and dysfunction in daily life caused by the disease can lead to emotions such as anxiety and depression (63), which can also affect the results of fMRI. Therefore, these factors should be included as covariates in future studies. Third, only patients with motor aphasia were included in this study. Future research is necessary to examine the effects of EA in more types of aphasia.

## Conclusions

In conclusion, EA at HT5 and GB39 may improve language function by modulating FC stability among the LN, SN, CN, occipital, somatosensory, and cerebral limbic system regions.

## Data availability statement

The raw data supporting the conclusions of this article will be made available by the authors, without undue reservation.

## Ethics statement

The studies involving human participants were reviewed and approved by the Medical Research Ethics Committee of Dongzhimen Hospital (ECPJ-BDY-2015-04) and all subjects gave written consent to participate. The patients/participants provided their written informed consent to participate in this study.

## Author contributions

YG and JC designed the study. TL, ZT, CL, XL, XH, and JX gathered and managed data. HZ and BZ provided guidance on experimental design and data analysis. MX was responsible for the manuscript's conception, methods, and writing. JC, QK, GK, and SL revised the manuscript. All authors contributed to the article and approved the submitted version.

## Funding

This work was supported by Dongzhimen Hospital (hospital/grants), and grants were supported by Special Project of International Cooperation of Traditional Chinese Medicine of State Administration of Traditional Chinese Medicine (project no. 0610-2140NF020630), Special Public Welfare Industry and Scientific Research from the State Administration of Traditional Chinese Medicine (project no. 201407001-9), and National Natural Science Foundation of China (project no. 81973790).

## Conflict of interest

The authors declare that the research was conducted in the absence of any commercial or financial relationships that could be construed as a potential conflict of interest.

## Publisher's note

All claims expressed in this article are solely those of the authors and do not necessarily represent those of their affiliated organizations, or those of the publisher, the editors and the reviewers. Any product that may be evaluated in this article, or claim that may be made by its manufacturer, is not guaranteed or endorsed by the publisher.

## Abbreviations

PSA: post stroke aphasia
HC: healthy control
EA: electroacupuncture
PPI: Psychophysiological Interaction
static ICA: static independent component analysis
dyn, ICA: dynamic independent component analysis
DMN: default mode network
SMN: sensorimotor network
CRRCAE: Chinese Rehabilitation Research Center for Chinese Standard Aphasia test
BDAE: Boston Diagnostic Aphasia Examination
VN: visual network
SN: salience network
DAN: dorsal attention network
FPN: frontoparietal network
LN: language network
CN: cerebellar network
AG: angular gyrus
SMG: supramarginal gyrus
PostCG: postcentral gyrus
ANTs: Advanced Neuroimaging Tools
