# Corrigendum: Modulations of static and dynamic functional connectivity among brain networks by electroacupuncture in post-stroke aphasia

**DOI:** 10.3389/fneur.2023.1148220

**Published:** 2023-02-10

**Authors:** Minjie Xu, Ying Gao, Hua Zhang, Binlong Zhang, Tianli Lyu, Zhongjian Tan, Changming Li, Xiaolin Li, Xing Huang, Qiao Kong, Juan Xiao, Georg S. Kranz, Shuren Li, Jingling Chang

**Affiliations:** ^1^Department of Neurology, Dongzhimen Hospital, Beijing University of Chinese Medicine, Beijing, China; ^2^Key Laboratory of Chinese Internal Medicine Ministry of Education, Beijing University of Chinese Medicine, Beijing, China; ^3^Institute for Brain Disorders, Beijing University of Chinese Medicine, Beijing, China; ^4^Department of Rehabilitation Sciences, The Hong Kong Polytechnic University, Hong Kong, Hong Kong SAR, China; ^5^The State Key Laboratory of Brain and Cognitive Sciences, The University of Hong Kong, Hong Kong, Hong Kong SAR, China; ^6^Department of Psychiatry and Psychotherapy, Comprehensive Center for Clinical Neurosciences and Mental Health, Medical University of Vienna, Vienna, Austria; ^7^Division of Nuclear Medicine, Department of Biomedical Imaging and Image-Guided Therapy, Medical University of Vienna, Vienna, Austria

**Keywords:** electroacupuncture, brain networks, post stroke aphasia, functional connectivity, psychophysiological interaction analysis, independent component analysis

In the published article, there was an inappropriate language error. The description of the EA technique omitted important details. A correction has been made to the section Stimuli and scanning procedure, Acupuncture procedures and needling sensation recording. The corrected paragraph is below.

Both the PSA and HC groups received EA stimulation at the HT5 and GB39 acupoints during fMRI acquisition. Before the start of the scan, the participants positioned themselves on the fMRI scanner bed on their backs, and needles were placed at the GB39 and HT5 acupoints. According to the “Name and Location of Acupoints” (GB/T 12346-2006), two acupoints were located on both sides. HT5 was situated radially to the flexor carpi ulnaris tendon on the anteromedial side of the forearm, 33 mm proximal to the palmar wrist crease, with insertion depths ranging from 10 to 30 mm. GB39 was needled at an insertion depth of 33 mm, 100 mm above the external malleolus tip, on the anterior fibula border (Figure 2A). A professional acupuncturist performed acupuncture. All participants reported their experience (“Deqi”) with acupuncture stimulation. Deqi featured aching, pressure, heaviness, fullness, and numbness among other feelings (23–29). The acupuncturist used 0.40 × 40-mm sterile silver acupuncture needles (Guizhou, China) with the EA technique. Han's acupoint nerve stimulator (model LH-202H) was situated outside the fMRI room, with one end of the acupoint wire linked to the acupuncture needle handle of the HT5 acupoint, and the other end connected to the acupuncture needle handle of the GB39 acupoint. The EA frequency was 2 Hz, and the electric current was 2 mA. As previously mentioned, the stimulation waveform is the dilatational wave (23).

The authors apologize for this error and state that this does not change the scientific conclusions of the article in any way. The original article has been updated.

