# Supplementary material for: Modulations of static and dynamic functional connectivity among brain networks by electroacupuncture in post-stroke aphasia
Source: Front Neurol. 2022 Dec 1;13:956931. doi: 10.3389/fneur.2022.956931 (PMC9751703; doi:10.3389/fneur.2022.956931)
Supplement: Supplementary file 1 [file Presentation_1.pdf]

Supplementary Material

1 Supplementary Figures and Tables

1.1 Supplementary Figures

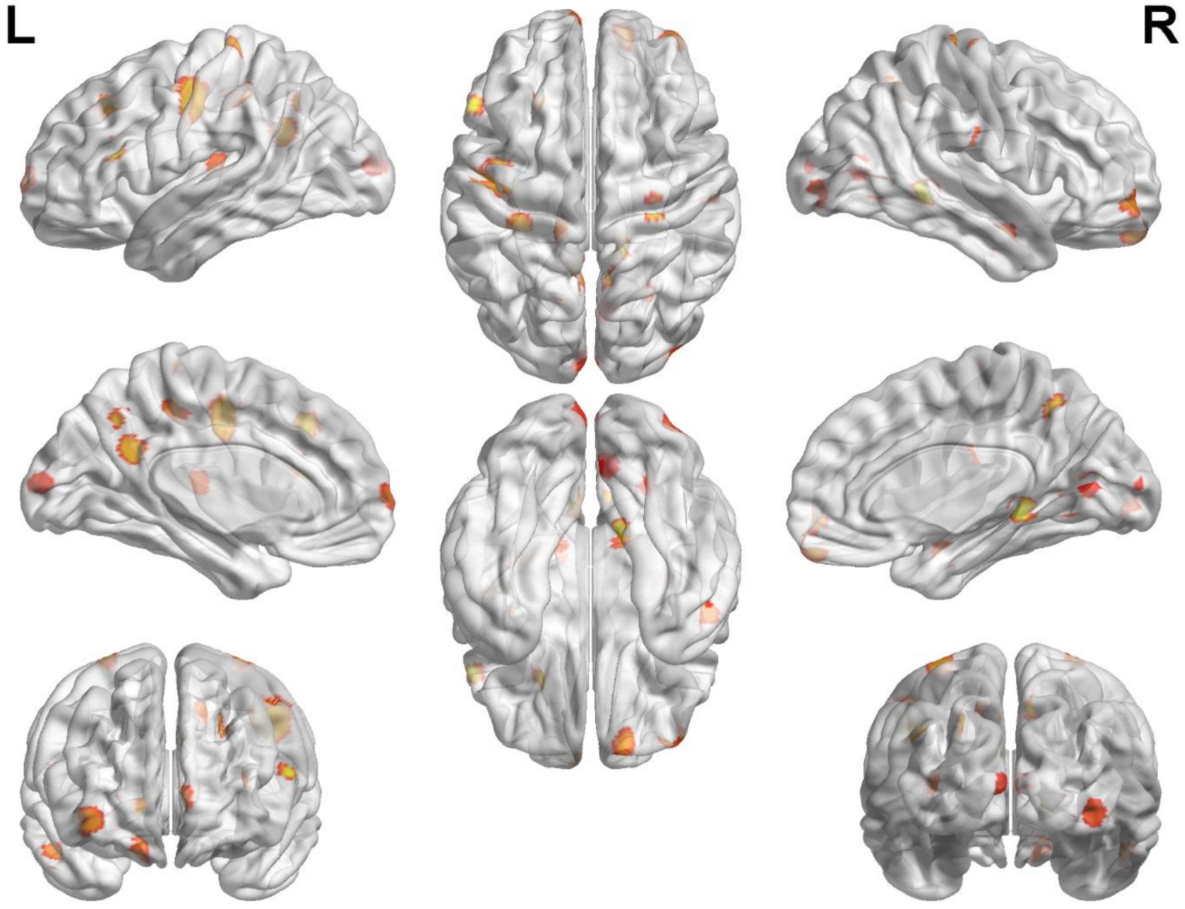

Supplementary Figure 1. The ROIs used in the ROI-to-ROI connectivity analysis. ROIs obtained from our previous multi-voxel pattern analysis (MVPA) results were used to create 6 mm radius spherical masks by WFU-Pick Atlas software.

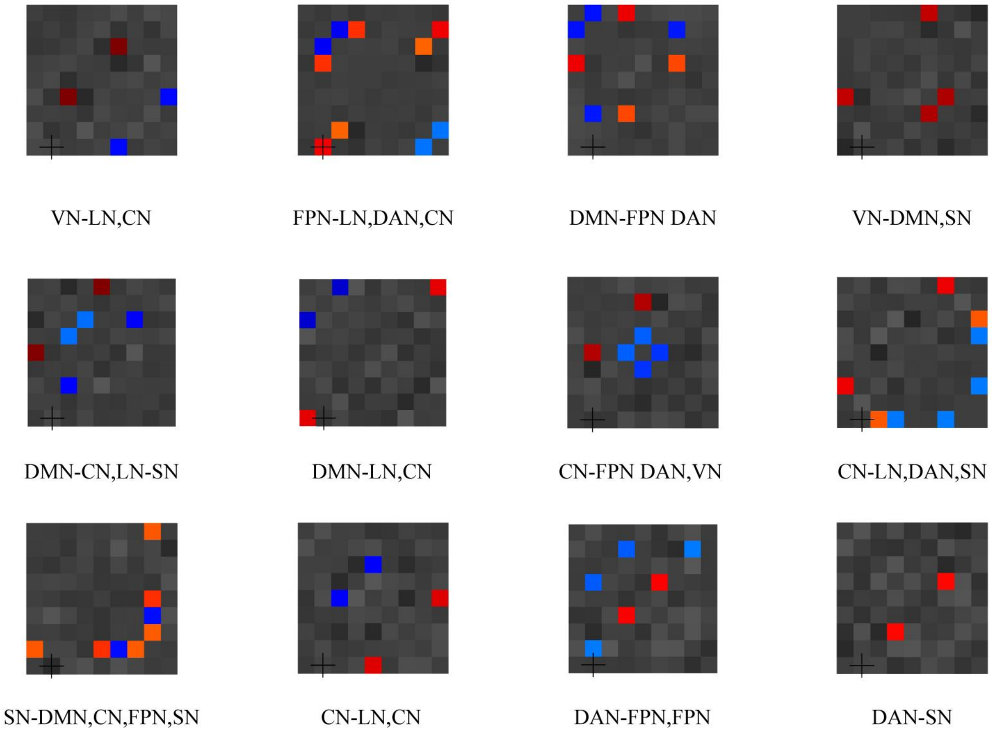

Supplementary Figure 2. A representation of the 12 identified dynamic factors named after the most prevalent between network interaction present in the dynamic factor.

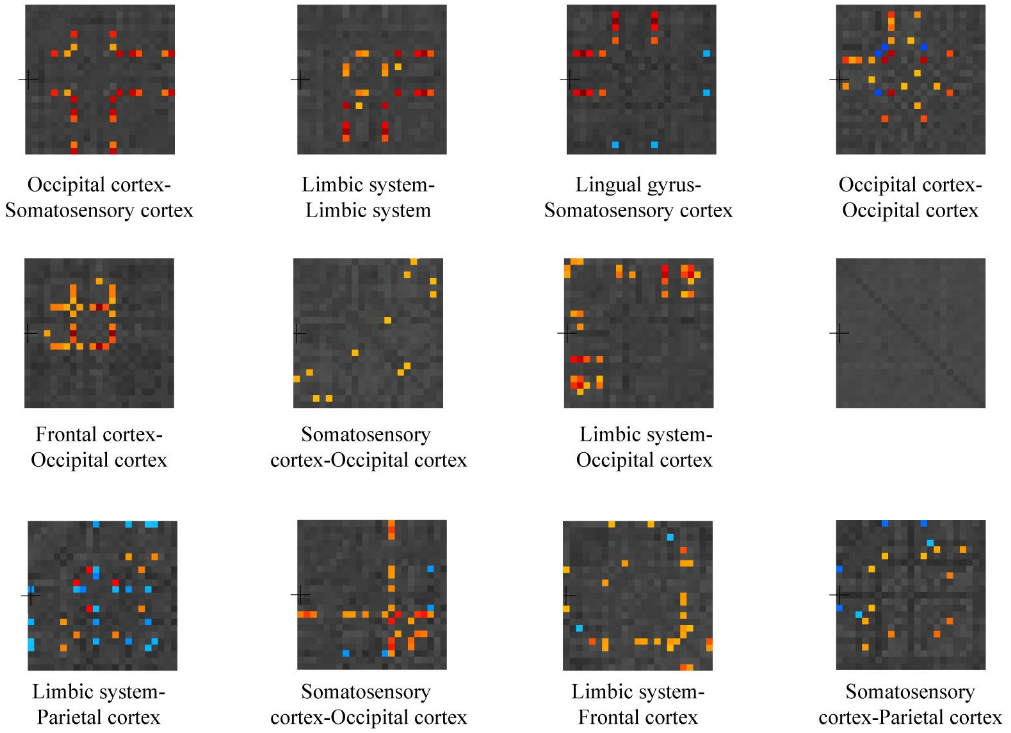

Supplementary Figure 3. A representation of the 12 identified dynamic factors named after the most prevalent between ROI interaction present in the dynamic factor.

1.2 Supplementary Tables

Table 1 Temporal properties varies within each group and condition

Component number	Component description	Frequency		Variability	
		F Statistics	P-FDR	F Statistics	P-FDR
IC1	DWN	1.01	0.320	-0.11	0.912
IC2	Noise	-1.49	0.146	0.22	0.993
IC3	FPN	0.75	0.462	0.22	0.825
IC4	LN	0.35	0.731	-0.85	0.403
IC5	DAN	-0.21	0.832	-1.12	0.271
IC6	CN	-2.93	**0.006**	-0.84	0.408
IC7	Noise	0.99	0.332	-1.94	0.062
IC8	Noise	0.68	0.503	-1.93	0.064
IC9	VN	-0.66	0.517	0.73	0.471
IC10	Noise	1.07	0.294	0.28	0.781
IC11	SN	0.36	0.722	-1.17	0.251
IC12	Noise	-0.04	0.968	1.18	0.249
IC13	Noise	-2.59	0.015	-1.11	0.276
IC14	Noise	-0.79	0.436	-1.05	0.301
IC15	SN	1.70	0.100	2.23	**0.034**
IC16	Noise	0.64	0.530	0.10	0.920
IC17	CN	-0.09	0.927	-0.83	0.412
IC18	Noise	2.91	0.007	-0.13	0.893
IC19	Noise	-1.72	0.097	0.44	0.663
IC20	Noise	0.17	0.865	0.35	0.730
